# Longitudinal Changes in the Myocardial T1 Relaxation Time, Extracellular Volume Fraction, and Left Ventricular Function in Asymptomatic Men

**DOI:** 10.3390/jcdd10060252

**Published:** 2023-06-09

**Authors:** Sang Hwa Shin, Sung Mok Kim, Soo-Jin Cho, Yeon Hyeon Choe

**Affiliations:** 1 Department of Radiology, Samsung Medical Center, Sungkyunkwan University School of Medicine, Seoul 06351, Republic of Korea; sh77.shin@samsung.com (S.H.S.); sm5040.kim@samsung.com (S.M.K.); 2 Health Promotion Center, Samsung Medical Center, Sungkyunkwan University School of Medicine, Seoul 06351, Republic of Korea; soojin77.cho@samsung.com

**Keywords:** myocardial T1 relaxation time, cardiac magnetic resonance imaging, extracellular volume fraction

## Abstract

(1) Background: Longitudinal changes in myocardial T1 relaxation time are unknown. We aimed to assess the longitudinal changes in the left ventricular (LV) myocardial T1 relaxation time and LV function. (2) Methods: Fifty asymptomatic men (mean age, 52.0 years) who underwent 1.5 T cardiac magnetic resonance imaging twice at an interval of 54 ± 21 months were included in this study. The LV myocardial T1 times and extracellular volume fractions (ECVFs) were calculated using the MOLLI technique (before and 15 min after gadolinium contrast injection). The 10-year Atherosclerotic Cardiovascular Disease (ASCVD) risk score was calculated. (3) Results: No significant differences in the following parameters were noted between the initial and follow-up assessments: LV ejection fraction (65.0 ± 6.7% vs. 63.6 ± 6.3%, *p* = 0.12), LV mass/end-diastolic volume ratio (0.82 ± 0.12 vs. 0.80 ± 0.14, *p* = 0.16), native T1 relaxation time (982 ± 36 vs. 977 ± 37 ms, *p* = 0.46), and ECVF (24.97 ± 2.38% vs. 25.02 ± 2.41%, *p* = 0.89). The following parameters decreased significantly from the initial assessment to follow-up: stroke volume (87.2 ± 13.7 mL vs. 82.6 ± 15.3 mL, *p* = 0.01), cardiac output (5.79 ± 1.17 vs. 5.50 ± 1.04 L/min, *p* = 0.01), and LV mass index (110.16 ± 22.38 vs. 104.32 ± 18.26 g/m^2^, *p* = 0.01). The 10-year ASCVD risk score also remained unchanged between the two timepoints (4.71 ± 0.19% vs. 5.16 ± 0.24%, *p* = 0.14). (4) Conclusion: Myocardial T1 values and ECVFs were stable over time in the same middle-aged men.

## 1. Introduction

Diffuse myocardial fibrosis can manifest in various diseases, such as hypertension, aortic stenosis, cardiomyopathy, myocarditis, diabetes mellitus, and heart failure [1,2]. The myocardial T1 relaxation time is highly correlated with diffuse myocardial fibrosis [2,3,4,5]. It aids the early detection of amyloidosis and Anderson—Fabry disease and also enables the assessment of myocardial damage severity following acute myocardial infarction [6,7,8]. T1 mapping enables the calculation of the extracellular volume fraction (ECVF), which is increased in conditions such as edema or inflammation [9,10].

T1 times and ECVF might change in relation to the progression of diseases or durations of disease, as mentioned above. However, threshold values for myocardial abnormalities have not been established according to age and sex to date. Furthermore, it has not been clearly determined whether alterations in T1 times or ECVF in follow-up examinations suggest disease progression and not a change due to aging.

Rosmini et al. revealed that T1 mapping techniques yielded inconsistent results regarding the native T1 time with respect to increasing age, and that the ECVF was independent of age for all mapping techniques [11]. However, longitudinal changes in the myocardial T1 times and ECVF within the same individual have not been examined previously. Therefore, our aim was to evaluate the longitudinal changes in the left ventricular (LV) myocardial T1 time and ECVF within the same asymptomatic individuals.

## 2. Materials and Methods

### 2.1. Patient Selection

Our Institutional Review Board Center approved this retrospective study. We evaluated the records of asymptomatic individuals who underwent cardiac magnetic resonance (CMR) imaging for the screening of cardiac diseases, including ischemic heart disease, at our institution between March 2012 and March 2021. We excluded the following subjects: (1) those who only underwent one test without a follow-up examination (*n* = 2528), (2) those lacking an optimal sequence for T1 mapping (*n* = 60), and (3) women (because of the very small sample size).

Thus, 50 asymptomatic men (mean age ± 1 standard deviation, 52.0 ± 5.3 years) were finally included in this retrospective study.

**Figure 1:**
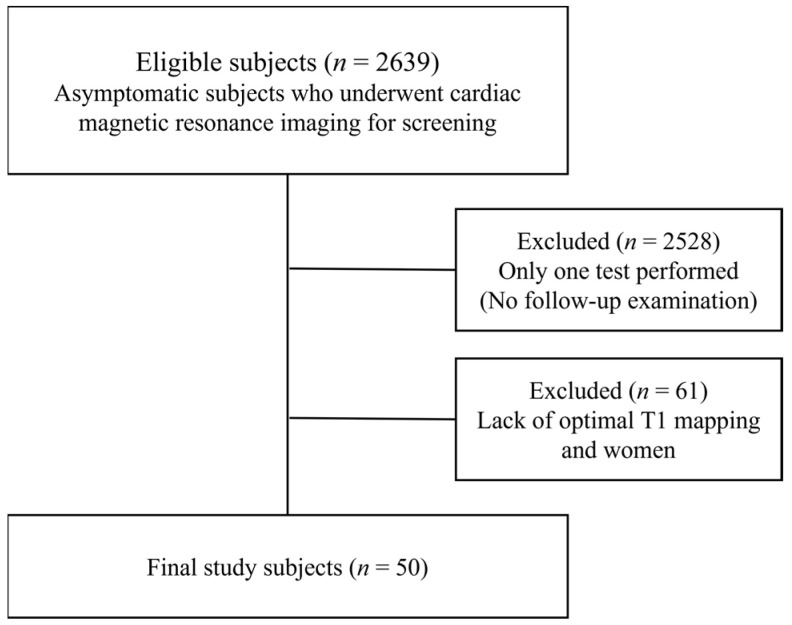
Flow chart depicting the inclusion of the study population. Asymptomatic subjects who underwent cardiac magnetic resonance imaging for screening at our institution were included. Subjects who underwent only one test without a follow-up examination (*n* = 2528), those whose examination lacked an optimal sequence for T1 mapping (*n* = 60), and women (*n* = 1) were excluded.

They underwent 1.5 T CMR imaging (Magnetom Avanto; Siemens Healthineers) with a 32-channel phased-array receiver coil twice at a mean interval of 54 ± 21 months (range, 11–112 months). The study selection process for the 2639 subjects screened is shown in Figure 1. The subjects chose to undergo CMR examinations as a health checkup, regardless of their cardiovascular risk factors, as CMR can help diagnose both ischemic and structural heart diseases without radiation hazard and with smaller amount of contrast materials. In addition, repeat CMR examinations were conducted again years later as part of the health checkup.

### 2.2. CMR Protocol and Image Analysis

**Figure 2:**
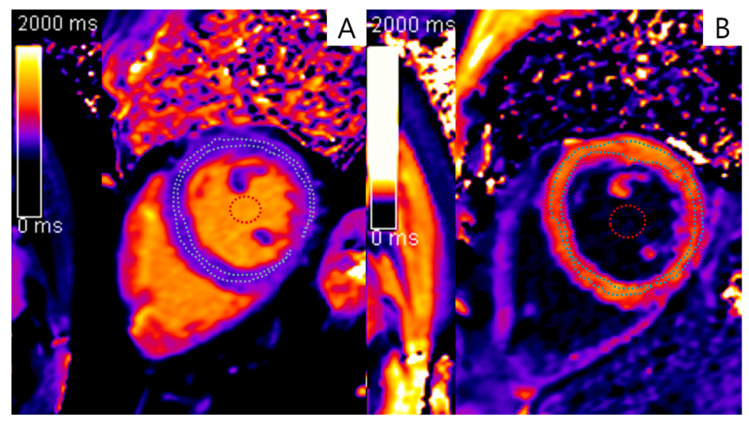
Short-axis slices at the mid-ventricle level of the pre-contrast and post-contrast reconstructed T1 maps acquired using the MOLLI technique. Regions of interest were manually drawn at the mid-ventricle level of the left ventricular myocardium (blue dotted line) and blood pools (red dotted line). (**A**) Before gadolinium contrast injection. (**B**) Fifteen min after gadolinium contrast injection.

All subjects were under fasting state without hydration for eight to ten hours before CMR examinations. Beta-blockers were not administered to them before CMR examinations. CMR scanning was performed using the MOLLI technique (before and 15 min after gadolinium contrast injection [0.1 mmol/kg of gadobutrol, Bayer Healthcare]). Image acquisition and analysis were performed in accordance with the consensus statement by the Society for Cardiovascular Magnetic Resonance [12]. Two cardiac radiologists who were experienced in CMR (one had 2 years of experience, while the other had 32 years of experience) calculated the LV native T1 and post-contrast T1 times in consensus by manually drawing the regions of interest (ROI) in the LV myocardium at the mid-ventricle level (Figure 2) using dedicated software (Syngo.via; Siemens Healthineers). The T1 relaxation time of the blood pool, required for ECVF calculations, was measured in the cardiac chamber at the same level while avoiding the papillary muscles. All original images were assessed for artifacts caused by susceptibility and cardiac or respiratory motion. Each motion-corrected series was evaluated for correct image alignment, and each map was evaluated to determine whether the original images were transformed into an acceptable map. Fully automated inline non-rigid motion correction was applied between the individual TI images before performing a T1 fit using a mono-exponential 3-parameter pixel-wise curve fit.

An observer with 13 years of experience in the data acquisition and analysis of CMR images analyzed the cardiac short-axis cine images (slice thickness, 6 mm; slice interval, 10 mm; cardiac phases, 30) using an Argus workstation (Siemens Healthineers) to calculate the global LV function parameters (LV ejection fraction, end-systolic volume, end-diastolic volume, mass, stroke volume, and cardiac output).

### 2.3. ECVF Calculation

The ECVF was calculated using the following formula:

(1 − hematocrit) × ∆R1myocardium/∆R1blood

where R1 = 1/T1.

The hematocrit values were obtained from venous blood samples collected on the same day that CMR imaging was performed [12].

### 2.4. Clinical Information

The Atherosclerotic Cardiovascular Disease (ASCVD) 2013 Risk Calculator (provided by the American Heart Association/American College of Cardiology [13]) was used to calculate the risk scores. This calculator is commonly used to estimate the risk of atherosclerotic cardiovascular disease development. It considers various risk factors, such as blood pressure, cholesterol levels, smoking status, and diabetes. In this study, age was excluded from the scoring because it was considered an independent variable.

### 2.5. Statistical Analysis

To compare the differences between the initial study and the follow-up study within the same subjects, we conducted a paired t-test. In the group difference analysis, we used an independent t-test to confirm the difference between the two groups. A simple correlation analysis using the Pearson correlation coefficient (r) was performed to examine the relationship between the T1 times and cardiovascular risk factors. Specifically, a correlation analysis was performed between the myocardial T1 times in the pre-contrast assessment and the following risk factors in the initial and follow-up assessments: age, risk score, LV ejection fraction, end-systolic LV volume (ESV), end-diastolic LV volume (EDV), LV mass index, and LV mass/end-diastolic volume ratio. Additionally, a correlation analysis was also performed on the difference between the myocardial T1 times or ECVF and the following risk factors: age, risk score, LV ejection fraction, ESV, EDV, LV mass index, and LV mass/end-diastolic volume ratio. Finally, changes in the T1 times and ECVF were correlated with changes in the LV function parameters, risk scores, and study duration. We performed a Bonferroni correction to adjust p-values to account for potential spurious significant results when probing for simple relationships with multiple variables.

Intra-observer and inter-observer variabilities in two observers were measured by calculating intraclass coefficients (ICCs) for LV function parameters, T1 times, and ECVF in 20 randomly-selected examinations for analyses at one-month intervals.

## 3. Results

The baseline characteristics of the 50 men and their status with respect to the cardiovascular risk factors are summarized in Table 1. Left ventricular function of the subjects at the initial and follow-up assessments are summarized in Table 2.

Significant late gadolinium enhancement, indicative of old myocardial infarction or cardiomyopathy, was not present in all subjects.

**Figure 3:**
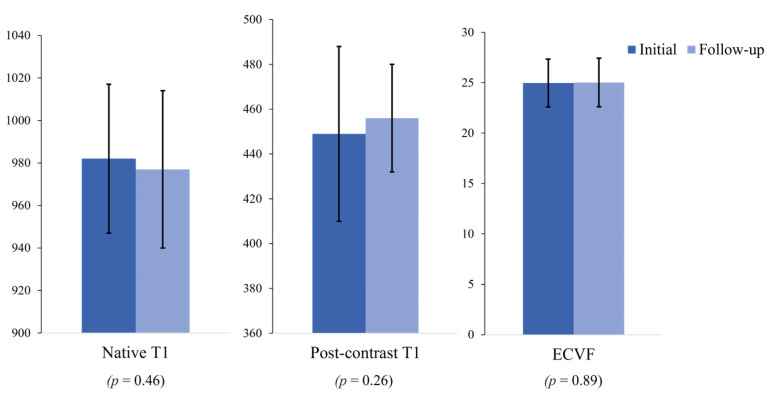
Mean T1 mapping values at the initial and follow-up assessments in 50 asymptomatic men. There was no significant change in the T1 mapping values, including the native T1 values, post-contrast T1 values, and ECVFs during follow-up. ECVF, extracellular volume fraction.

No significant differences were noted between the initial and follow-up assessments with respect to the LV ejection fraction (65.0 ± 6.7% vs. 63.6 ± 6.3%, *p* = 0.12), LV mass/end-diastolic volume ratio (0.82 ± 0.12 vs. 0.80 ± 0.14, *p* = 0.16), native T1 times (982 ± 36 vs. 977 ± 37 ms, *p* = 0.46), post-contrast T1 times with 15 min delay (449 ± 39 vs. 456 ± 24 ms, *p* = 0.26), and ECVF (24.97 ± 2.38% vs. 25.02 ± 2.41%, *p* = 0.89) (Figure 3). However, the following parameters decreased significantly from the initial assessment to the follow-up: stroke volume (87.2 ± 13.7 mL vs. 82.6 ± 15.3 mL, *p* = 0.01), cardiac output (5.79 ± 1.17 L/min vs. 5.50 ± 1.04 L/min, *p* = 0.01), and LV mass index (110.16 ± 22.38 g/m^2^ vs. 104.32 ± 18.26 g/m^2^, *p* = 0.01). The 10-year ASCVD risk score did not change significantly during follow-up (initial vs. follow-up: 4.71 ± 0.19% vs. 5.16 ± 0.24%, *p* = 0.14).

**Figure 4:**
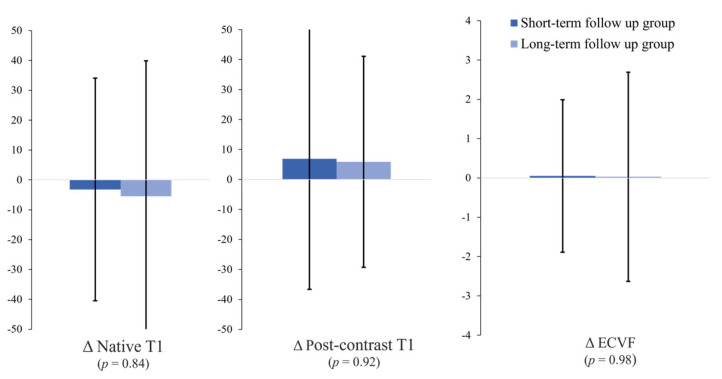
Comparison of the T1 values and ECVFs between the short-term follow-up group and the long-term follow-up group. No significant differences are noted in these parameters between the two groups. Error bars represent 1 standard deviation.

Group difference analysis using the follow-up interval based on the mean follow-up interval of 54 months (Table 3 and Figure 4) revealed no significant differences between the short-term follow-up group (<54 months, *n* = 27) and the long-term follow-up group (≥54 months, *n* = 23) with respect to the change (Δ) in the native T1 mapping values (−3.2 ± 37.3 vs. −5.5 ± 45.4 ms, *p* = 0.84), Δpost-contrast T1 value (6.9 ± 43.5 vs. 5.9 ± 35.2 ms, *p* = 0.92), and ΔECVF (0.05 ± 1.94 vs. 0.03 ± 2.66, *p* = 0.98).

**Figure 5:**
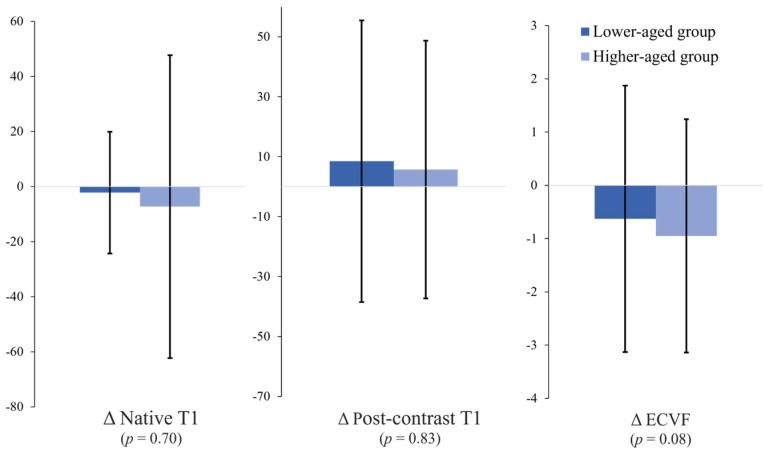
Comparison of the T1 values and ECVFs between the lower-aged group and the higher-aged group. No significant differences are noted in these parameters between the two groups. Error bars represent 1 standard deviation.

Group difference analysis by age based on the mean age of 52 years revealed no significant differences between the lower-aged group (age < 52 years, *n* = 29) and the higher-aged group (age ≥ 53 years, *n* = 21) with respect to the Δnative T1 value (−2.2 ± 26.1 vs. −7.3 ± 55.9 ms, *p* = 0.70), Δpost-contrast T1 value (8.5 ± 47.1 vs. 5.7 ± 43.0, *p* = 0.83), and ΔECVF (−0.63 ± 2.50 vs. 0.95 ± 2.19, *p* = 0.08) (Figure 5).

Results of the analysis of the correlation between the myocardial T1 values/ECVF and the risk factors are summarized in Table 4. Similarly, results of the analysis of the correlation between changes in the myocardial T1 values/ECVF and the cardiovascular factors or left ventricular function parameters are summarized in Table 5. Significant negative correlations were observed between the ECVF and age (*p* = 0.006), and EDV (*p* < 0.001) at the initial assessment. No significant correlation was observed between the myocardial T1times and the cardiovascular risk factors and LV function parameters at the initial and follow-up assessments. Additionally, no significant correlation was observed between the myocardial ECVF and the cardiovascular risk factors and LV function parameters at the follow-up assessments.

Correlation analysis showed a significant negative correlation (r = −0.47) between changes in the myocardial T1 time and changes in the EDV (*p* < 0.001), while the correlations were insignificant between other LV function parameters and changes in T1 times or ECVF.

No adverse cardiac events were reported until the dates of follow-up CMR examinations in all subjects.

ICCs for intra-observer variability were 0.98, 0.99, and 0.98 for LVESV, LVEDV, and LV mass, respectively, and ICCs for inter-observer variability for them were 0.95, 0.88, and 0.98, respectively. ICCs for inter-observer variability were 0.95 and 0.88 for T1 mapping and ECVF, respectively, and ICCs for intra-observer variability for them were 0.95 and 0.95, respectively.

## 4. Discussion

Our study demonstrated that no significant changes occurred in the myocardial T1 time and ECVF over time in asymptomatic men, regardless of their age or follow-up interval. The LV ejection fraction and LV mass/end-diastolic volume ratio did not differ significantly between the initial and follow-up assessments. However, the stroke volume, cardiac output and LV mass decreased significantly during the follow-up period. A significant negative correlation was observed between changes in the myocardial T1 time and changes in the EDV.

Physiological factors, such as age, sex, weight, height, body mass index, hematocrit, and heart rate, may affect myocardial T1 mapping [14,15]. Previous studies in humans have shown a consistent correlation between the myocardial T1 value and the extent of diffuse myocardial fibrosis quantified using endomyocardial biopsy [16,17]. Diffuse myocardial fibrosis is known to have an accelerated progression in many diseases, including hypertension, aortic stenosis, cardiomyopathy, myocarditis, diabetes mellitus, and heart failure [1,9,10]. Previous studies have investigated the correlation between age and T1 values. However, to the best of our knowledge, no research has been conducted on serial T1 mapping within the same asymptomatic individuals.

According to the Multi-Ethnic Study of Atherosclerosis (MESA) by Rosmini et al. [11], Kim et al. [18], Rauhalammi et al. [19], and Piechnik et al. [14], the T1 time and ECVF were higher in women than in men; conversely, Dabir et al. reported no correlation between age, sex, and the native T1 value [20]. Similarly, in healthy African-Americans, the native T1 time at 3 T scanning was not influenced by age, sex, and body mass index [21].

In some studies, the T1 time decreased with increasing age in women, whereas the ECVF was not influenced by aging. Rosmini et al. reported that the native myocardial T1 time decreased slightly, while the ECVF remained unaffected, with increasing age [11]. According to Rauhalammi et al., the mean native T1 time decreased with increasing age in healthy women aged <33 years; however, it did not vary with age in men [19]. In the MESA study, linear regression analyses demonstrated that greater partition coefficients, pre-contrast T1 values, and ECVs were associated with older age in men. The ECVF was also significantly associated with age in women after multivariable adjustment.

In a study on healthy individuals, Piechnik et al. observed no age dependency of the myocardial T1 values in individual and age group-based regression analyses [14]. In their study, the myocardial T1 time was consistently higher in women than in men up to the age of 45 years; thereafter, a convergence was noted, with no significant differences between the two sexes. The T1 time did not vary with age in both sexes in other studies as well [14,18,20,21]. In a study on asymptomatic individuals, the T1 value and ECVF did not differ significantly among four age groups divided into quantiles [18]; no significant linear correlation was observed between age and the T1 value or ECVF in the total population and in each sex group.

In one study, the T1 values were similar across the age groups regardless of the field strength used (1.5 T vs. 3 T) [20]. In another study, the global native T1 time decreased by 5.50 ms for each 10-year rise in the age; however, this was only at 1.5 T [19]. Dabir et al. also reported that the T1 values were similar across the age groups for all field strengths tested (group 1: ≤30 years [n, 1.5 T = 27, 3 T = 26], group 2: 31–42 years [n, 1.5 T = 28, 3 T = 27]; group 3: 42–53 years [n, 1.5 T = 27, 3 T = 24], group 4: ≥53 years [n, 1.5 T = 28, 3 T = 28]). A trend of positive association was observed between the native T1 time and age at 1.5 T (r = 0.21, *p* < 0.1 for all) but not at 3 T; this association was stronger in the male cohort (r = 0.23, *p* = 0.04).

As for the left ventricular function parameters, according to a meta-analysis of normal reference ranges, men have larger EDV and ESV indices and a higher LV mass than women [22]. The LV ejection fraction does not differ significantly between men and women. EDV indices are larger in younger individuals (20–40 years) than in older individuals (≥65 years). The LV mass is lower among East Asians (Chinese, Korean, and Singaporean-Chinese) than among Caucasians. Furthermore, the LV mass index of Brazilian men and women is greater than that of East Asians; however, it is lower than that of Caucasians [22]. A significant relationship was also observed between a lower LV mass index and 3 T scanners. In the MESA study, the LV ejection fraction was higher in women than in men, while the LV mass, volume, stroke volume (all indexed according to the body surface area), and mass-to-volume ratio were higher in men than in women [15]. According to the study by Davir et al., native T1 showed a mild association with LV-EDV, LV-ESV, and LV mass at both 1.5 T and 3 T field strengths [20].

According to our analysis, stroke volume and cardiac output were found to decrease with aging. One study reported a significant relationship between age and cardiac output, showing a reduction of approximately 1 percent per year [23]. They explained that the decline in cardiac output observed in their study was attributed to a decrease in stroke index associated with decreases in body size and heart rate due to aging. Additionally, another paper indicated that vagal withdrawal may account for increased heart rate during the initial minute of exercise in younger individuals, the diminished responsiveness to beta-adrenergic stimulation is primarily responsible for the reduced maximal heart rate and cardiac output in the elderly [24].

Only age and LVEDV were seen to be correlated negatively with ECVF at the initial assessment, while ASCVD risk scores, LVEF, LV mass, ESV, and LV mass/EDV ratio were not correlated with T1 times or ECVFs. Again, only changes in LVEDV were seen to be negatively correlated with changes in T1 values. Our study results suggest that most LV function parameters do not change over time regardless of changes in T1 times or ECVF in asymptomatic middle-aged men in less than 5 years. According to a meta-analysis [22], both men and women in younger age had significantly larger indexed LVEDV, which may be in line with the finding of a negative correlation between changes in T1 times and changes in LVEDV in our study.

This study had several limitations. First, it was performed retrospectively at a single center. Therefore, our findings should be validated in a larger prospective study. Second, due to the low rate of examinations in women, our study only included men. The higher native T1 time and ECVF in women than in men in several studies suggest the influence of sex hormones on the myocardial structure and function [11,14,15,18,19]. Third, T2 relaxation times were not quantified and analyzed in this study.

## 5. Conclusions

The myocardial T1 relaxation times and ECVF were stable over time in the same individuals. Changes in the EDV were negatively correlated with changes in the myocardial T1 times.

## Figures and Tables

**Table 1:** Clinical characteristics of the study subjects at the initial and follow-up assessments.

Characteristics	Initial Assessments	Follow-Up Assessments
Age (years)	52.0 ± 5.3	56.4 ± 5.6
Hematocrit (%)	43.4 ± 3.2	44.1 ± 2.8
Heart rate (beats/min)	66.4 ± 10.9	67.2 ± 11.2
Total cholesterol (mg/dL)	184.8 ± 34.5	181.2 ± 38.7
HDL cholesterol (mg/dL)	55.8 ± 14.1	56.8 ± 14.1
Triglyceride (mg/dL)	136.2 ± 102.6	128.6 ± 87.4
Creatinine (mg/dL)	0.96 ± 0.13	0.91 ± 0.14
Body weight (kg)	71.9 ± 10.8	72.4 ± 11.2
BMI (kg/m^2^)	24.2 ± 2.4	24.3 ± 2.7
Systolic blood pressure (mmHg)	121.5 ± 31.9	120.3 ± 17.0
Diastolic blood pressure (mmHg)	76.1 ± 12.4	77.4 ± 11.9
Treated for high blood pressure	13 (26 %)	18 (36 %)
Diabetes	2 (4 %)	3 (6 %)
Treated for diabetes	1 (2%)	3 (6%)
Smoking	8 (16 %)	8 (16 %)
10-year ASCVD risk (%)	4.71 ± 0.19	5.16 ± 0.24

There were no significant changes in the cardiovascular risk factors or 10-year ASCVD risk during the interval between the initial and follow-up assessments (*p* = 0.14). ASCVD, atherosclerotic cardiovascular disease; BMI, body mass index; HDL, high-density lipoprotein.

**Table 2:** Left ventricular function of the subjects at the initial and follow-up assessments.

Left Ventricular Measurements	Initial Assessments	Follow-Up Assessments	*p*-Value
LV ejection fraction (%)	65.0 ± 6.7	63.6 ± 6.3	0.12
LV mass/end-diastolic volume ratio	0.82 ± 0.12	0.80 ± 0.14	0.16
LV end-systolic volume (mL)	46.3 ± 10.4	47.1 ± 10.4	0.60
Systolic volume index (mL/m^2^)	24.7 ± 4.7	25.3 ± 4.9	0.16
LV end-diastolic volume (mL)	133.7 ± 18.2	135.6 ± 42.3	0.76
Diastolic volume index (mL/m^2^)	72.2 ± 8.1	73.7 ± 24.4	0.33
Stroke volume (mL)	87.2 ± 13.7	82.6 ± 15.3	0.01
Stroke volume index (mL/m^2^)	47.4 ± 6.8	44.6 ± 6.5	0.01
Cardiac output (L/min)	5.7 ± 1.1	5.5 ± 1.0	0.01
Cardiac index (L/min/m^2^)	3.1 ± 0.5	2.9 ± 0.4	0.01
LV mass index (g/m^2^)	110.2 ± 22.4	104.3 ± 18.3	0.01

The stroke volume, cardiac output, and LV mass index decreased significantly during the interval. The other parameters did not show any significant changes. LV, left ventricle.

**Table 3:** Differences in the T1 mapping values and extracellular volume fractions between the short-term follow-up and the long-term follow-up groups.

	Short-Term Follow-Up Group (<54 Months, *n* = 27)	Long-Term Follow-Up Group (≥54 Months, *n* = 23)	*p*-Value
Δnative T1 value (ms)	−3.2 ± 37.3	−5.5 ± 45.4	0.84
Δpost-contrast T1 value (ms)	6.9 ± 43.5	5.9 ± 35.2	0.92
ΔECV fraction (%)	0.05 ± 1.94	0.03 ± 2.66	0.98

There was no significant difference in the ΔT1 mapping values and extracellular volume fractions between the short-term follow-up and long-term follow-up groups. ECV, extracellular volume.

**Table 4:** Analysis of the correlation between the myocardial T1 values or extracellular volume fractions and the cardiovascular risk factors or left ventricular function parameters at the initial assessment.

Analysis of the Correlation between the Myocardial T1 Values at the Initial Assessment and the Risk Factors and LV Function Parameters	Correlation Coefficient (r)	*p*-Value
Age	−0.14	0.60
ASCVD risk score	−0.10	>0.95
LVEF	−0.13	0.70
LV mass	−0.18	0.38
EDV	−0.17	0.42
ESV	0.08	>0.95
LV mass/end-diastolic volume ratio	−0.08	>0.95
Analysis of the Correlation between the ECVF at the Initial Assessment and the Risk Factors and LV Function Parameters	Correlation Coefficient (r)	*p*-Value
Age	−0.41	0.006
ASCVD risk score	−0.30	0.074
LVEF	−0.12	0.74
LV mass	0.01	>0.95
EDV	−0.54	<0.001
ESV	0.07	>0.95
LV mass/end-diastolic volume ratio	0.24	0.14

There was a significant negative correlation between the ECVF and the age, and EDV at the initial assessment. EDV, end-diastolic volume; EF, ejection fraction; ESV, end-systolic volume.

**Table 5:** Analysis of the correlation between changes in the myocardial T1 value or ECVF and the cardiovascular risk factors or left ventricular function parameters.

Analysis of the Correlation between Changes in the Myocardial T1 Values and the Risk Factors and LV Function Parameters	Correlation Coefficient (r)	*p*-Value
Study interval	0.28	0.08
ΔASCVD risk score	−0.21	0.26
ΔLVEF	0.04	>0.95
ΔLV mass	0.02	>0.95
ΔEDV	−0.47	<0.001
ΔESV	0.07	>0.95
ΔLV mass/EDV ratio	0.26	0.12
Analysis of the Correlation between Changes in the ECVF and the Risk Factors and LV Function Parameters	Correlation Coefficient (r)	*p*-Value
Study interval	0.05	>0.95
ΔASCVD risk score	0.08	>0.95
ΔLVEF	−0.17	0.44
ΔLV mass	0.14	0.62
ΔEDV	−0.17	0.40
ΔESV	0.18	0.36
ΔLV mass/EDV ratio	0.27	0.1

Significant negative correlation was found between changes in the myocardial T1 values and changes in the EDV.

## Data Availability

All data underlying the results are available as part of this article and no additional source data are required.
